# A moderated-mediation analysis of performance appraisal politics perception and counterproductive work behavior

**DOI:** 10.3389/fpsyg.2022.928923

**Published:** 2022-10-25

**Authors:** Hong-Yan Wang, Zhi-Xia Chen

**Affiliations:** ^1^College of Economics and Management, Hubei Polytechnic University, Huangshi, China; ^2^College of Public Administration, Huazhong University of Science and Technology, Wuhan, China

**Keywords:** performance appraisal politics perception (PAPP), perceived organizational justice, individual political skill, counterproductive work behavior (CWB), social comparison theory (SCT)

## Abstract

Politics has become a common element in the performance appraisal process, and as decision recipients in this process, those appraised tend to be more sensitive to performance appraisal politics. This paper examines the mechanisms by which performance appraisal politics perception (PAPP) affects counterproductive work behavior (CWB) from the perspective of those appraised. The mediating effect of perceived organizational justice (POJ) and the moderating effect of political skill (PS) are incorporated into a parsimonious moderated-mediation model. A quantitative research approach is employed with survey data from 460 employees of large and medium-sized enterprises in Hubei Province (China), and structural equation modeling (SEM) and bootstrap analysis are used to test the proposed hypothesized relationships. The findings demonstrate that PAPP has a positive impact on CWB, and POJ partly mediates the relationship between PAPP and CWB. The results also reveal that individual PS moderates the positive correlation between PAPP and CWB. The academic and practical implications of these findings, as well as limitations and suggestions for future research, are also discussed.

## Introduction

Over the past few decades, the study of performance appraisal has attracted interest, and abundant research indicates that the most important feature of an ideal performance appraisal system is the accuracy of its rating results (Tsai and Wang, 2013; Iqbal et al., 2015; Raveendran and Hameela, 2020; Rubin and Edwards, 2020). One of the most extensively studied non-performance features of such systems—and one of the contextual determinants of performance rating accuracy—is the political use of rating (Adler et al., 2016; Lavigne, 2018; Tziner and Rabenu, 2018). There is growing evidence that inaccuracy in the rating is more likely to be caused by the deliberate and volitional distortion of performance appraisal than previously believed unintentional deviation (Tziner and Rabenu, 2018). “Any realistic discussion of performance appraisal must recognize that organizations are political entities” (Longenecker et al., 1987, p 184); as such, appraisers are political actors who view the performance appraisal as no more than a discretionary tool for motivating and rewarding subordinates and the reasonable use of power in a more flexible way (Dhiman and Maheshwari, 2013). Appraisers thus often attempt to use the appraisal process to their advantage (Lonckgen, 1989; Tziner and Rabenu, 2018). The ambiguity and instrumentality of the performance appraisal process thus drive participants to indulge in this kind of political manipulation (Ferris and Judge, 1991; Poon, 2004), and we should therefore admit that performance appraisal politics are an inevitable reality of organizational life (Tziner and Rabenu, 2018).

Performance appraisal politics has become a common part of the process (Longenecker et al., 1987), so it is important to know how such politics influences employees’ reactions and behaviors. The mainstream view holds that performance appraisal politics exerts a negative effect on employees’ attitudes and behaviors, including turnover intention (Javed et al., 2013; Chaudhry et al., 2016; Rosen et al., 2017; Imran, 2019; Imran et al., 2019; Naseeb et al., 2019; Tabassum et al., 2021); employee performance (Imran et al., 2019; Tabassum et al., 2021); and deviant work behavior (Nagi et al., 2022). Although valuable studies exist, these findings pay too much attention to the appraiser’s perspective and attach great importance to the appraiser’s political behavior, while inadequate attention has been paid to the perceptions of appraisees. As decision recipients, appraisees have a “higher instrumental stake in the decision than appraisers, so they experience appraisal politics more acutely” (Dhiman and Maheshwari, 2013, p 1,204). According to Dhiman and Maheshwari (2013), performance appraisal politics perception (PAPP) is appraisees’ perception of “the manipulative actions by appraisers and appraisees to influence ratings to achieve their self-serving performance appraisal goals” (p 1,205). Appraiser rating manipulation dominates performance appraisal politics, but, importantly, the fellow appraisees’ actions related to political involvement and appraisal-linked organization decisions must not be ignored, especially as coworkers’ “upward influence” may change appraisers’ political considerations. As such, it should be noted that the raters are not the sole factor determining performance appraisal politics. The appraisee puts the focus not only on the appraiser’s political behavior but also on the political activities of the coworkers and the organization’s political considerations (e.g., promotion, pay). Indeed, appraisee PAPP reflects the complexity of appraisal politics relating to the multi-faceted social interaction among appraisers, appraisees, and the organization, and the influence of performance appraisal politics is not likely to be one-sided (i.e., from the supervisor to the subordinate).

The most relevant research is currently oriented toward the relationship between performance appraisal politics and employee turnover intention, while little attention has been paid to other negative organizational behaviors such as counterproductive work behavior (CWB), which is defined as any intentional actions threatening or harming the legitimate norms and interests of an organization or its members (Gruys and Sackett, 2003; Spector et al., 2006). As a long-standing dilemma at the workplace, CWB has widespread and destructive consequences for both organizations and individuals (Cohen and Diamant, 2019; Cohen and Özsoy, 2021; Searle, 2022). It is particularly noteworthy that the negative impact of CWB rates on organizational performance is stronger than the positive impact of employee positive behavior rates (Dunlop and Lee, 2004), so it is worthwhile to investigate the antecedents of CWB. Although studies have examined the influence of organizational politics (Makhdoom et al., 2017; Sajjad et al., 2017; Meisler et al., 2020; Habib et al., 2022) and organizational justice (Makhdoom et al., 2017; Cohen and Diamant, 2019; Mahadiputra and Piartrini, 2021; Mursita and Nahartyo, 2022) on employee CWB, there are merely fragmented studies on the relationship between performance appraisal justice and CWB (Colquitt and Zipay, 2015; Cohen and Diamant, 2019; Cohen and Özsoy, 2021). No in-depth investigations on the relationship between performance appraisal politics and CWB have been conducted.

Manipulative behaviors in the performance appraisal system can be subsumed under the heading of organization politics (Tziner and Rabenu, 2018). Instead of focusing on the wider aspects of organizational politics, a targeted discussion of the effect of performance appraisal politics on CWB is meaningful due to the targeted efficacy of tailored interventions over non-tailored ones. The current research is thus based on the appraisee perspective and explores the association between PAPP and CWB, while discussing the mediating role of organizational justice in this relationship. Not all appraisees with high PAPP may implement CWB, and this relationship is likely to be somewhat dependent on individual competencies or characteristics (e.g., political skill, PS). Every organization is actually a political arena (Mintzberg, 1983), and employees should possess heightened levels of PS “to effectively navigate these turbulent waters” (Ferris et al., 2003, p 406). From the perspective of organizational politics, the researcher views PS as individual characteristics that are sets of adaptability-enhancing abilities within the context of organizational politics (Mintzberg, 1983). Politically skilled employees have a highly developed self-regulatory capacity (García-Chas et al., 2015; Xue et al., 2020), as well as the ability to achieve goals by influencing those around them (Kranefeld et al., 2020). Prior studies have confirmed that individual PS significantly alleviates the negative outcomes of organizational politics (Sajjad et al., 2017; Crawford et al., 2019; Bhattaral, 2021; Atshan et al., 2022; Kaur and Kang, 2022) and is helpful for the appraisee to achieve more positive supervisor ratings in the performance appraisal process (Kwon, 2020).

Nevertheless, cultural idiosyncrasies need to be considered before implementing any best practices (Dhiman and Maheshwari, 2013), and attention must be given to cultural variability at the organizational level within each nation (DeNisi and Smith, 2014; Tziner and Rabenu, 2018). Face is not a unique cultural phenomenon found only in China, but it has long been essential for understanding general Chinese psychology and behavior (Song, 2018). Saving face is considered to be essential in collectivistic cultures because the harmony of collectivistic cultures is the process whereby face is regulated in a given social structure (Merkin, 2018). Maintaining harmony with others is thus the core of PS in collectivist culture (Lin and Sun, 2012; Fang et al., 2015; Kwon, 2020; Wu et al., 2020). But saving the face of another is also an important capability within the set of PS for Chinese employees in a collectivist culture because the harmony and interdependence of collectivistic cultures necessitate concern for the other or mutual “face-saving” (Fang et al., 2015; Lu and Guy, 2018; Merkin, 2018). This is consistent with Confucius’s view of humaneness. Appraisees’ high PS may present more salient strategic advantages in the appraisal game within collectivist cultures (Merkin, 2018; Song, 2018; Kwon, 2020), so it is important to explore the role of individual PS in the effects of PAPP on CWB, with especial consideration given to how to positively activate PS to tackle the complex appraisal politics situation in a Chinese culture background.

In the current research, we tease out a demarcated set of variables relevant to PAPP, including perceived organization justice (POJ), PS, and CWB, all of which have been associated with PAPP. In doing so, this study strives to answer the following essential and interconnected research questions:

RQ1. Does PAPP have an influence on CWB?

RQ2. Does POJ mediate the association between PAPP and CWB?

RQ3. Does PS moderate the relationship between PAPP and CWB?

The present study adds to the existing body of literature in three key ways. First, it systematically explores the inner psychological mechanism of PAPP on CWB from the appraisee’s perspective. To the best of our knowledge, no prior studies have done this, so our research fills this gap. Previous studies have focused more on the appraiser perspective rather than the appraisee perspective; this examines the appraiser’s political behavior, while the appraisee perspective takes into account the political behavior of coworkers and the organization beyond the appraiser. Unlike existing studies, which have used social exchange theory to interpret the foundations of performance appraisal politics affecting employee attitude and behavior, we assert that social comparison theory (SCT) may provide an additional explanation for how PAPP affects CWB. This enriches the theoretical basis for illustrating the regular influence of performance appraisal politics in organizations.

Second, the existing literature has suggested that PAPP is culture-driven (Dhiman and Maheshwari, 2013; Hofman and Newman, 2014). To the best of our knowledge, little is known about whether performance appraisal politics are appropriate or effective in the Chinese collectivist cultural setting or how they affect the behaviors of appraisees and appraisers in such circumstances. A collectivist cultural setting would be fertile ground for practices that manipulate appraisal politics (Kwon, 2020), so we sought to explore the cultural properties of performance appraisal politics through the psychological and behavioral tendencies of Chinese employees, and make several contributions to the existing body of knowledge by considering the subject in a non-Western setting.

Third, a previous study has verified the positive effect of PS on supervisor ratings (Kwon, 2020), but to date, no study has examined its moderating effect on performance politics, and little research has investigated how appraisees use PS to cope with the negative consequences of performance appraisal politics. Higher PS among appraisees may present more salient strategic advantages in the appraisal game for a collectivist culture (Kwon, 2020). Moreover, the harmony and interdependence of collectivistic culture necessitate other-concern or mutual “face-saving” (Fang et al., 2015; Lu and Guy, 2018; Merkin, 2018). Thus, in addition to the general property of PS proposed, we claim that face business is also an important PS for Chinese employees.

In this study, we discuss the role of appraisee PS in the relationship between PAPP and CWB, which extends the literature on PS, performance politics, and CWB. To sum up, the findings should be of benefit to organizations seeking to master the mechanisms through which performance appraisal politics influence employee attitude and behavior while providing a profound perception of the importance of understanding appraisal politics in specific cultural settings. This can also help organizations determine how to enhance the accuracy and effectiveness of performance appraisals given the political context. The paper begins with a review of the key themes in the research and then explores the literature on PAPP that might be linked to CWB. Following the presentation of the hypotheses, the data are described in more detail. The study then turns to empirical analysis. Finally, the paper concludes by reflecting on the implications of the findings and outlining steps for future studies.

## Theoretical background and hypotheses

### Theoretical discussion

According to SCT, individuals often assess their abilities or status in an organization by comparing themselves to those with similar abilities (Festinger, 1954). Social comparison is a widespread human disposition (Whitaker et al., 2018), and is an aspect of performance appraisal. In the performance appraisal process, appraisers often ignore typical “absolute” rating standards and prefer to compare an employee with others (Goffin et al., 2009). Appraisees, meanwhile, are not only concerned about the accuracy of their own ratings (Cohen and Diamant, 2019), but also compare their ratings with the performance results of fellow employees (Colquitt and Zipay, 2015). Employees, therefore, decide what to do or what to consider by comparing their ratings with coworkers who have similar social characteristics. When employees develop the belief that their performance ratings are determined by political considerations rather than their own performance—and even find that the ratings of coworkers with similar abilities may be higher than theirs—due to this disadvantaged social comparison, a feeling of organizational injustice may be evoked, which in turn causes negative behavior, such as CWB. Setting aside the use of social exchange theory to interpret the employee reaction to the appraiser’s manipulation, we claim the influence of the appraisee’s PAPP on CWB is achieved through a process of social comparison in which emotional or perception experiences emerge and in turn trigger corresponding behavior.

### Performance appraisal politics perception and counterproductive work behavior

As mentioned above, CWB refers to any deliberate, intentional employee behavior that is detrimental to the legitimate interest of an organization as well as its members (Gruys and Sackett, 2003; Spector and Fox, 2010; Schwager et al., 2016). CWB includes absenteeism, disobeying orders, reducing output, showing offensive acts, and even theft or substance abuse (Spector et al., 2006; Cohen and Diamant, 2019). CWB exists broadly in various organizations, and its negative effects are appreciably more pronounced as the size and life cycle of the organization grow (Chirumbolo, 2015). Some researchers have found that employees’ perception of the fairness of and their satisfaction with performance appraisal have a negative relationship with CWB (Colquitt and Zipay, 2015; Cohen and Diamant, 2019). Some studies have also explored the profound effect of performance appraisal politics on employee organizational behavior (Rosen et al., 2017; Imran, 2019; Naseeb et al., 2019; Tabassum et al., 2021; Nagi et al., 2022), but less attention has been paid to the influence of performance appraisal politics on CWB.

According to SCT, individuals have a fundamental desire to accurately evaluate their abilities and define themselves through objective and non-social standards, but when appraisal standards are inaccurate or unavailable, individuals tend to evaluate themselves by comparison with others (Festinger, 1954). Performance appraisal is the basis of a reward system that directly affects employees’ positive attitudes as well as their behaviors (Imran et al., 2019) and helps employees to identify their contributions and status in the workplace. If ratings are manipulated by political considerations (e.g., power, interpersonal relationships) rather than the employee’s real performance (Dhiman and Maheshwari, 2013), appraisees are inclined to evaluate their status and success based on social comparisons with others such as their coworkers. When appraisees discover themselves to be disadvantaged in these social comparisons, they are particularly inclined to enact CWB to balance their discontent. Accordingly, the first hypothesis is proposed as follows:

H1: Employee’s PAPP has a positive influence on CWB.

### Mediating role of perceived organizational justice

Fairness has been demonstrated to be one of the classic elements affecting appraise reactions to performance appraisals (Iqbal et al., 2015; Tziner and Rabenu, 2018). Perceived organizational justice (POJ) refers to the employee’s perception that he or she is treated fairly within an organization (Greenberg, 1990; Pattnaik and Tripathy, 2018). Organizational justice is a multidimensional construct that consists of distribution, procedural, and interaction justice (Yean, 2016; Pattnaik and Tripathy, 2018; Mursita and Nahartyo, 2022). Studies have confirmed that CWB could be seen as a reaction to perceived injustice and primarily related to an employee changing his/her input to restore equity (Makhdoom et al., 2017; Cohen and Diamant, 2019; Mahadiputra and Piartrini, 2021; Mursita and Nahartyo, 2022). When employees perceive distributive injustice, they may make the outcome/input ratio less negative by engaging in CWB such as withdrawal, sabotage, resistance, and theft (Pan et al., 2018); deviant behaviors (Shoaib and Baruch, 2019); or refusal to follow instruction (Yean, 2016). The social comparison process is integral to the origin of organizational justice (Koopman et al., 2020). Performance appraisal is inherently comparative and easily evokes a sense of injustice in terms of both procedures and rewards (Dhiman and Maheshwari, 2013; Shoaib and Baruch, 2019). When appraisees perceive that the standard of appraisal distribution, procedures, and interactions are manipulated by political considerations (e.g., power, *guanxi*, *renqing*), the comparison becomes more significant for them. Studies have also established a negative correlation between organizational politics and injustice (Khattak et al., 2020; Mursita and Nahartyo, 2022). Therefore, the correlations among organizational justice, PAPP, and CWB are interrelated based on SCT, but currently, such links have not been investigated. Therefore, we present the following hypotheses:

Hypothesis H2a: PAPP is negatively correlated with POJ.

Hypothesis H2b: Employee POJ is negatively correlated with CWB.

Hypothesis H2c: Employee POJ mediates the relationship between PAPP and CWB.

### Moderating role of individual political skill

The successful harmony of employees requires many skills and abilities to fit the work, including PS (Al-Abrrow et al., 2019; Atshan et al., 2022). PS refers to the individual ability to effectively understand the intentions and behaviors of others and to modify personal behavior to meet the needs and requirements of the surrounding work environment (Mintzberg, 1983; Ferris et al., 2008). Individuals with relatively high PS scores are characterized by social astuteness, interpersonal influence, networking ability, and apparent sincerity (Templer, 2018; Kwon, 2020). PS relieves the negative reactions of employees such as pressure and tension (Munyon et al., 2015), emotional labor (Sajjad et al., 2017), job dissatisfaction (Atshan et al., 2022), and knowledge hiding (Kaur and Kang, 2022), and it also makes the employee more likely to achieve positive supervisor ratings. When appraisees with high PS are inclined to harmonize with others and value face, they seem to excel in utilizing their PS to deal with the performance appraisal politics (Kwon, 2020) and “to effectively navigate these turbulent waters” (Ferris et al., 2003, p 406). This allows them to cope with organizational justice without difficulty. In contrast, appraisees with a low level of PS have insufficient political abilities and may have difficulty responding flexibly to confront performance appraisal politics, so their POJ tends to be negative. Consequently, this study proposes the following hypotheses:

H3a: The level of PS moderates the relationship between PAPP and POJ such that the relationship is weaker for employees with high PS than for those with low PS.

Based on the hypotheses, we propose a moderated-mediation hypothesis, namely that the level of the appraisee’s PS negatively moderates the indirect effect of PAPP on CWB *via* POJ. Specifically, a high level of PS leads to a stronger indirect effect of PAPP on CWB *via* POJ. In comparison, a low level of PS leads to a weaker indirect effect of PAPP on CWB *via* POJ. Therefore, we postulated the following:

H3b: The indirect effect of PAPP on CWB *via* POJ will be weaker when the level of PS is high rather than low.

**FIGURE 1:**
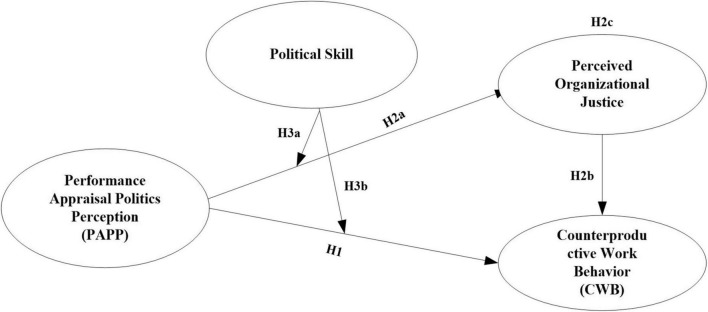
The theoretical hypothesis model.

Thus, based on the above theoretical foundations, we derived the theoretical framework for this research, as shown in Figure 1.

## Methodology

### Participants and procedure

Data were obtained from a survey questionnaire that was circulated and distributed among employees in seven large and medium-sized enterprises in Wuhan and Huangshi, Hubei province, China, during July and August 2020. Participants were all employed on a full-time basis and randomly selected by the human resource manager in each enterprise. All participants provided written informed consent for participation and were guaranteed anonymity; they were also informed that their employers would only be provided with the aggregate findings of the research.

In an attempt to overcome the problems of common method bias and consistent with Jordan and Troth’s (2020) recommendations, we provided clear instructions to participants about how to complete the questionnaire, kept the questionnaire concise, varied the anchor labels on the scales, and separated the location of the independent and dependent variables. To do this, each participant completed the questionnaire twice at an interval of 2 weeks. Information related to participants’ demographic characteristics, PAPP, and PS was collected through interviews in the first survey. The second survey was conducted 2 weeks after the first survey with the same participants from the first survey, and the information about participants’ POJ and CWB was collected *via* an online survey questionnaire.

A total of 542 questionnaires were collected, with a total of 460 usable responses received (85%). Of the 460 questionnaires, males accounted for 77.6% and females for 22.4%; their average age was 35.20. In terms of education level, participants with college or associate degrees or below accounted for 26.20%, those with a bachelor’s for 62.50%, and master’s and above for 11.30%. Regarding job tenure (years), 10.54% of respondents had been in their position for 3 years or less, 41.82% for 3–5 years, 38.17% for 5–10 years, and 9.47% for 10 years or more.

### Measurement

The questionnaires adopted in this research were well-established and commonly used in the literature. We carefully followed the standard procedure of back-translation, and asked participants to rate the extent of PAPP, POJ, PS, and CWB using five-point Likert scales ranging from 1 (strongly disagree) to 5 (strongly agree).

#### Performance appraisal politics perception

The measurement of PAPP developed by Dhiman and Maheshwari (2013) was used to measure the appraisee’s subjective perception of performance appraisal politics. This scale contains three dimensions: appraiser’s politics (five items), coworker politics (three items), and pay and promotion politics (five items). An example item is: “In my organization reward (pay and promotion) policies are politically applied.” The overall Cronbach’s alpha for PAPP was 0.83.

#### Perceived organizational justice

POJ was measured with Niehoff and Moorman’s (1993) 20-item scale, which was used to describe how employees are treated in the workplace. This scale contains three dimensions of organizational justice: procedural justice, distributive justice, and interpersonal justice. An example item is: “I consider my workload to be quite fair.” This scale has been widely used to evaluate organizational justice in previous studies (Shkoler et al., 2021). The overall Cronbach’s alpha for POJ was 0.88.

#### Political skill

Given the cultural variability in PS measures (Fang et al., 2015; Lu and Guy, 2018), we adopted the Chinese version of the PS questionnaire developed by Lin and Sun (2012). This scale was revised based on the version of Ferris et al. (2008), and used to assess Chinese employees’ level of PS based on the following five dimensions: interpersonal harmony (three items), social astuteness (five items), face-saving (four items), trickery use (three items), and networking ability (four items). An example item is: “I will compliment and praise the others in public to save their face.” The overall Cronbach’s alpha for PS was 0.94.

#### Counterproductive work behavior

CWB was measured with a 19-item scale developed by Bennett and Robinson (2000), which contained items related to organizational deviance and interpersonal deviance. An example item is: “I deliberately worked slower than I could.” The overall Cronbach’s alpha for CWB was 0.96.

#### Control variables

Following previous studies, this study controlled for demographic variables such as employee gender (0 = male, 1 = female); age (1 = 20 years old or less, 2 = 20–30 years old, 3 = 30–40 years old, 4 = 40–50 years old, 5 = 30 years old or more); education level (1 = college or associate’s degree or less, 2 = bachelor’s degree, 3 = master’s degree or above); and job tenure (1 = 3 years or below, 2 = 3–5 years, 3 = 5–10 years, 4 = 10 years or above).

### Statistical analysis

Descriptive statistics including means, standard deviations, and bivariate correlations were analyzed using SPSS 22.0. Pearson’s correlations were measured among PAPP, POJ, CWB, and PS to verify the associations among the variables, constructs, demographic (gender, age, education level), and work-related variables (years of job tenure). To test for common method bias, Harman’s single-factor test was applied in SPSS 22.0. Confirmatory factor analysis (CFA) (Kline, 2011) was performed to examine the measurement model with AMOS 20.0. Three item parcels were created for all the administered measures, to enhance our model’s reliability and parsimony. Each parcel was created by sequentially summing items assigned based on the highest to lowest item-total corrected correlations. Parceling made it possible to obtain fewer free parameters to estimate and reduce sources of sampling errors (Coffman and MacCallum, 2005; Little et al., 2013). The robust maximum likelihood approach (MLR) was used to deal with non-normality in the data (Wang and Wang, 2012).

Structural equation modeling (SEM) makes it possible to examine the degree to which a hypothesized model agrees with the observed data and also facilitates the simultaneous consideration of associations among latent constructs and observed variables in a model, as well as indirect effects while taking into account covariates. In this study, the moderated-mediation model presented in Figure 1 (including an independent variable, PAPP; a dependent variable, CWB; a mediator variable, POJ; and a moderator variable, POJ) was tested using SEM in AMOS20.0. Missing data were handled with full information maximum likelihood estimation. Following Sinacore (1993), all independent and moderating variables included were mean-centered to deal with multicollinearity. Path analysis was conducted to examine the associations among PAPP, POJ, CWB, and PS. To test for mediating effects, the bias-corrected bootstrap analysis method provides the most accurate confidence interval (CI) estimation and has the highest statistical efficacy (Hayes, 2013). In the current study, bootstrapping analysis was conducted to detect the significance of the estimated path in the mediation model based on 5,000 resamples for a 95% bias-corrected CI, using the SPSS macro PROCESS (with PAPP as the independent variable; CWB as the dependent variable; POJ as the mediator; and gender, grade, education level and job tenure as covariates). Bias-corrected bootstrapping is an often-recommended method for testing mediation due to its higher statistical power relative to other tests, and the indirect effect was considered statistically significant if the 95% bias-corrected CI did not contain zero (Hayes, 2013).

The model fit was evaluated with several goodness-of-fit indices (Schweizer, 2010): the goodness-of-fit index (GFI), the normed fit index (NFI), the adjusted goodness-of-fit index (AGFI), the comparative fit index (CFI), and the incremental fit index (IFI), with values > 0.90 indicating satisfactory fit and values > 0.95 good fits; the root mean square error of approximation (RMSEA), with values of < 0.08 indicating acceptable fit (Hu and Bentler, 1999); and the chi-square ratio by degrees of freedom (χ^2^/df), with values ≤ 5.0 indicating acceptable quality indicators of adjustment (Marôco, 2014). The average variance extracted (AVE) is a summary indicator of convergence, and an AVE of at least 0.50 means that the variance explained by the construct is greater than the measurement error (Fornell and Larcker, 1981). Effect sizes of the regression coefficients were evaluated by interpreting their standardized estimates (β); β-values of 0.10 were considered small, values of 0.30 were medium, and values of ≥ 0.50 were large (Cohen, 2013).

## Results

### Common method bias and measurement validity

Common-method variance bias was assessed with Harman’s single-factor test (Tehseen et al., 2017). Findings showed that 10 characteristic values emerging from the EFA were greater than l, accounting for 65.12% of the total variance, and a single factor only accounted for 24.33% of the covariance among the measures (Podsakoff and Organ, 1986), meaning that there were no issues associated with common method variance in the data.

CFA was used to measure the reliability of each of the items and constructs, and AVE scores were also assessed for each construct to test convergence validity. The results (see Table 1) of the CFA indicated that all fitness indicators exceeded the accepted threshold values, suggesting the model fit the data well. The AVE values for each latent variable were above 0.5, indicating good convergent validity. The results of the reliability and validity testing indicated that SEM was appropriate for testing the model.

**TABLE 1:** Confirmatory factor analysis of each variable.

Variables	AVE	GFI	AGFI	CFI	TLI	χ^2^/df	RMSEA
Performance appraisal politics perception	0.68	0.92	0.90	0.94	0.94	2.63	0.08
Perceived organizational justice	0.69	0.97	0.92	0.98	0.98	2.96	0.08
Political skill	0.58	0.92	0.91	0.94	0.94	2.11	0.07
Counterproductive work behavior	0.56	0.92	0.90	0.98	0.97	1.36	0.04

A theoretical model with four factors (PAPP, POJ, PS, and CWB) was assessed by CFA. The model fit indices are reported in Table 2, and the hypothetical four-factor model was quite acceptable (x^2^/df = 2.35, CFI = 0.95, NFI = 0.91, IFI = 0.94, RMSEA = 0.06).

**TABLE 2:** Model fit summary for hypothetical model and alternative models.

Model	χ^2^/df	RMSEA	CFI	NFI	IFI	GFI
Hypothetical four-factor model M1:PAPP, POJ, PS, CWB	2.35	0.06	0.95	0.91	0.94	0.96
Alternative three-factor model M2:PAPP + POJ = 1 factor, PS, CWB	2.67	0.07	0.91	0.90	0.95	0.94
Alternative three-factor model M3:PAPP + PS = 1 factor, POJ, CWB	5.28	1.32	0.99	0.97	1.00	1.00
Alternative two-factor model M4:PAPP + POJ + PS = 1 factor, CWB	0.77	0.37	1.00	0.99	1.00	1.00
Alternative two-factor model M5:POJ + PS + CWB = 1 factor, PAPP	31.34	0.35	1.00	1.00	1.00	1.00
Alternative one-factor model M6:PAPP + POJ + PS + CWB = 1 factor	36.11	0.41	1.00	1.00	1.00	1.00

*N* = 460. PAPP, Performance appraisal politics perception; POJ, perceived organizational justice; PS, political skill; CWB, counterproductive work behavior.

### Descriptive statistics and correlations

Means (*M*), standard deviations (*SD*), and bivariate correlations for all of the variables included in the SEM are summarized in Table 3. Descriptive analyses with SPSS 22.0 were based on *M* and *SD*. Preliminary analyses were carried out to test the relationships between the predictor (PAPP), and outcome (CWB) variables. Independent-sample *T*-test results indicated that the participants’ demographic characteristics (gender, age, education level, and job tenure) did not affect CWB. Pearson’s correlation analysis was used to explore the bivariate association between measured variables, and a *P*-value < 0.05 was defined as the level of significance. As shown in Table 3, PAPP was found to be negatively related to the mediator, namely POJ (*r* = −0.16, *p* < 0.05), while PAPP was positively related to the dependent variable, that is, CWB (*r* = 0.23, *p* < 0.01), and the moderating variable, that is, PS (*r* = −0.20, *p* < 0.01). POJ was also found to be linked to CWB (*r* = 0.40, *p* < 0.01).

**TABLE 3:** Descriptive and bivariate correlations analysis.

Variables	*M*	*SD*	1	2	3	4	5	6	7	8
Gender	1.35	0.02	–							
Age	2.13	0.64	0.15**	–						
Education level	2.76	0.43	0.03	0.04	–					
Job tenure	3.05	0.79	0.03	0.04	0.01	–				
PAPP	2.74	0.71	0.07	0.07	0.03	0.06	−			
POJ	3.54	0.76	0.01	0.05	0.02	0.05	−0.16*	–		
PS	3.66	0.70	0.06	0.05	0.03	0.05	−0.20**	−0.11*	–	
CWB	1.98	0.63	0.06	0.05	0.06	0.04	0.23**	−0.40**	−0.16*	–

*N* = 460. PAPP, Performance appraisal politics perception; POJ, perceived organizational justice; PS, political skill; CWB, counterproductive work behavior.

*p < 0.05; **p < 0.01.

### Hypothesis testing

**FIGURE 2:**
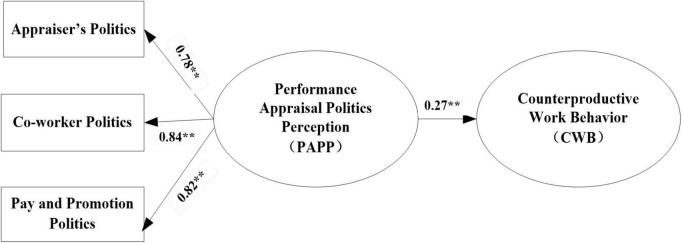
The model of direct relationship between PAPP and CWB. *N* = 460, **p < 0.01.

In this study, the moderated-mediation model presented in Figure 1 was tested using a three-step SEM strategy based on maximum likelihood estimation with AMOS 20.0. In Step 1 (model 1), the baseline model was used to examine the direct effect of PAPP→CWB for adequate fit. We tested the direct effect of PAPP on CWB, and the result (see Figure 2) showed that PAPP had a positive relation with CWB (β = 0.27, *p* < 0.01). The model provided a good fit to the data: χ^2^/df = 2.30, GFI = 0.95, NFI = 0.92, IFI = 0.95, TLI = 0.94, CFI = 0.95, RMSEA = 0.04. Therefore, hypothesis H1 (employee PAPP has a positive influence on CWB) was supported.

**FIGURE 3:**
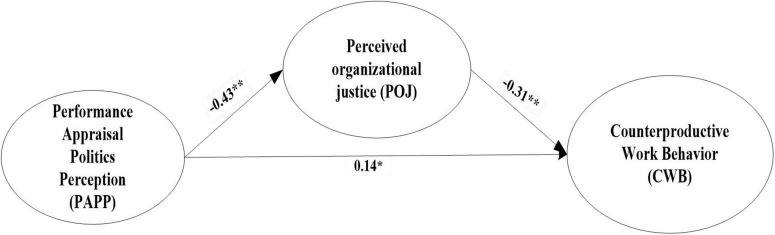
The mediating model of perceived organizational justice. *N* = 460, **p < 0.01, *p < 0.05.

In step 2 (model 2), the construct POJ was added to the baseline model. This was used to test the PAPP→CWB→POJ model for adequacy of fit according to Holmbeck’s (1997) three-step procedure for identifying mediation in SEM. Assuming adequate fit, the models PAPP→CWB, PAPP→CWB, and CWB→POJ were examined for significance. We then assessed the fit of the mediation model (PPAP→CWB→POJ) when (a) the PAPP→CWB path was constrained to zero, and (b) the PAPP→CWB was unconstrained. If the unconstrained model did not fit better than the constrained model, the PAPP→CWB model was reduced to non-significance, and full mediation was concluded. The results shown in Figure 3 indicate that PAPP was a significant predictor of POJ (β = −0.43, *p* < 0.01), while POJ negatively affected CWB (β = −0.31, *p* < 0.01); thus, hypotheses H2a and H2b were supported. Meanwhile, the findings also indicated that the positive relationship between PAPP and CWB was still significant (β = 0.14,*p* < 0.05), suggesting that POJ partly mediated the relationship between PAPP and CWB. The tested model had a good fit index (χ^2^/df = 2.27, GFI = 0.90, NFI = 0.91, IFI = 0.95, TLI = 0.94, CFI = 0.95, RMSEA = 0.05). Furthermore, the bootstrap analysis showed that the indirect effect of PAPP on CWB through POJ was significant (β = 0.13, *SE* = 0.09, *95% CI* = [0.04, 0.23], excludes zero), and the mediation effect (PPAP → POJ →CWB) accounted for 48% of the total effect, so hypothesis 2c was supported.

**FIGURE 4:**
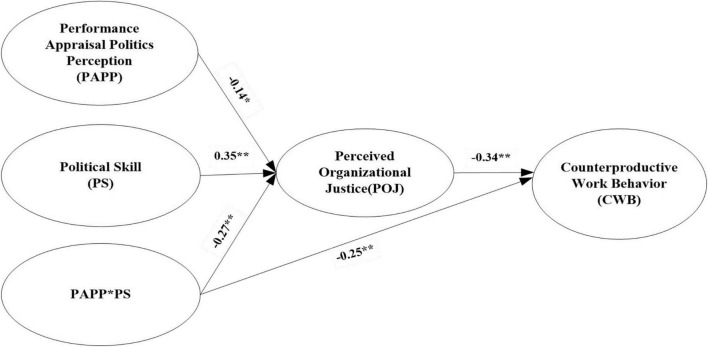
The Moderating model of political skill. *N* = 460, **p < 0.01, *p < 0.05.

In Step 3 (model 3), PS was added to the mediation model to examine the effects of PS on the relationship between PAPP and CWB. In Figure 4, the moderating model containing the independent (PAPP), dependent (CWB), and moderator (PS) variables, as well as the interaction variables (PAPP*PS) was within the acceptable fit (χ^2^/df = 2.69, GFI = 0.90, NFI = 0.93, TLI = 0.95, CFI = 0.99, RMSEA = 0.06). The results revealed that interaction variables (PAPP*PS), as predictors, were negatively associated with POJ (β = −0.27, *p* < 0.01), while the negative relationship between POJ and CWB was still significant in SEM (β = −0.34, *p* < 0.01). This indicated that the negative moderating effect of PS in the relationship between PAPP and POJ was significant. Hypothesis 3a was thus supported.

Meanwhile, the interaction variables (PAPP*PS) exhibited a significant negative effect on CWB (β = −0.25, *p* < 0.01), implying that PS negatively moderated the PAPP–CWB link, such that the higher the level of PS, the weaker the relationship between PAPP and CWB. The PROCESS macro with 5,000 resamples was used to generate bootstrap CIs for the conditional indirect effect of PAPP on CWB *via* POJ at different levels of employee PS. For an employee with a high level of PS, PAPP had a significant indirect effect on CWB through POJ [β = 0.32, *SE* = 0.08, *95%CI* = (0.17, 0.48), excludes zero]; however, this indirect effect became insignificant [β = 0.14, *SE* = 0.01, *95%CI* = (−0.04, 0.25), contains zero], when the employee PS level was low. The pairwise contrasts between these conditional indirect effects were significant [β = 0.09, *SE* = 0.03, *95%CI* = (0.04, 0.15), excludes zero]. Consequently, Hypothesis 3b was supported.

**FIGURE 5:**
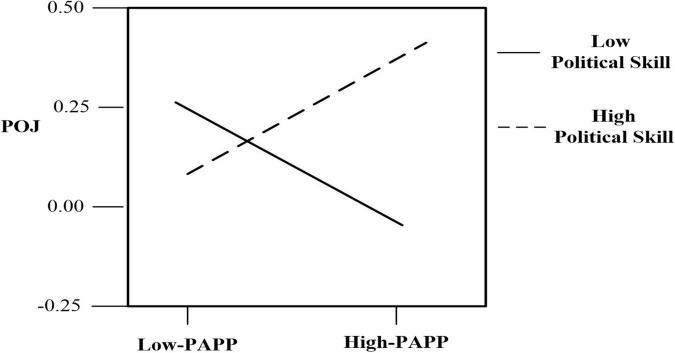
The Moderating effect of political skill on the relationship between PAPP and POJ.

To further investigate the moderating effects of PS in greater detail, regression equations were plotted at different levels of skill (i.e., one standard deviation above and below the mean; see Sinacore, 1993). The results of the equations are presented graphically in Figure 5. As shown, the negative relationship between PAPP and POJ was weaker as the level of PS increased, and employees with a high level of PS exhibited higher levels of POJ, regardless of the level of PAPP.

## Discussion

### Conclusion

As decision recipients, appraisees have a “higher instrumental stake in the decision than appraisers, so they experience appraisal politics more acutely” (Dhiman and Maheshwari, 2013, p 1,204). Thus, to some extent, the appraisees’ attitudinal and behavioral reactions to performance appraisal politics are more closely linked to their subjective perceptions than to objective reality. In the current research, we, therefore, investigated the main effects of appraisee PAPP on CWB and analyzed the mediating role of POJ in this association. We further introduced individual PS as a moderating variable in the association of PAPP with CWB.

Our study indicates that appraisee PAPP is positively correlated with CWB. That is, the appraisee who perceives a high level of performance appraisal politics in the workplace is prone to implement CWB. These findings are consistent with previous research showing a link between performance appraisal politics and employees’ negative behavior (Naseer and Ahmad, 2016; Rosen et al., 2017; Sajjad et al., 2017; Imran, 2019; Imran et al., 2019; Tabassum et al., 2021; Nagi et al., 2022). Although previous studies have confirmed that performance appraisal fairness and satisfaction are negatively associated with CWB (Colquitt and Zipay, 2015; Cohen and Diamant, 2019), little research has considered the influence of performance appraisal politics as an antecedent of CWB. Our research thus fills this gap and enlarges the literature on CWB.

The results also reveal that POJ partly mediates the relationship between PAPP and CWB. As such, when the appraisees realize that their ratings are manipulated in the organization, they are inclined to carry out CWB to alleviate organizational injustice. This finding is in line with the opinions that organization injustice evokes employee CWB (Makhdoom et al., 2017; Cohen and Diamant, 2019; Mahadiputra and Piartrini, 2021; Mursita and Nahartyo, 2022), but previous studies have not verified the correlation among performance appraisal politics, organization justice, and CWB. A collectivist cultural setting would be fertile ground for manipulative practices in appraisal politics because this setting attaches great importance to social norms and group harmony rather than individual performance (Kwon, 2020). In this case, the Chinese employees deeply influenced by collectivism preferred to ensure their status in the group through the social comparison process, which is a widespread practice (Whitaker et al., 2018; Ohdaira, 2019) frequently correlated with POJ (Koopman et al., 2020). According to SCT, the Chinese appraisees in the performance appraisal politics setting may assess their own abilities by comparing their ratings to those of similar coworkers. When they feel that their ratings are shaped by political manipulation rather than their real performance, or if there is an excessive gap between their ratings and those of others, POJ declines and is accompanied by CWB. For this reason, we believe the mediating mechanism of PAPP and CWB is based on SCT.

The present study also shows that PS negatively moderates the negative relationship between PAPP and CWB *via* POJ, which is in line with prior findings that PS is an effective individual characteristic to ensure survival in organizational political setting (Sajjad et al., 2017; Crawford et al., 2019; Bhattaral, 2021; Atshan et al., 2022; Kaur and Kang, 2022). The successful harmony of employees requires many skills and abilities to fit the work, including PS (Al-Abrrow et al., 2019; Atshan et al., 2022). High PS among appraisees presents more salient strategic advantages in the appraisal game for a collectivist culture (Kwon, 2020). An employee in the Chinese collectivist culture is inclined to pursue harmony with others, so they take full advantage of their PS to maintain overall harmony with coworkers in the workplace. Saving face is also seen as the other important competency in understanding the psychology and behavior of Chinese people (Song, 2018). In our study, we used the Chinese version of the PS construct proposed by Lin and Sun (2012), which has been revised based on Ferris et al. (2008) and takes into account both the general and special characteristics of PS. Face-saving is added to the Chinese version as a unique dimension.

### Theoretical implications

The theoretical contributions of this research are primarily reflected in three ways. First, it addresses a gap in the literature and recommends CWB as the outcome for performance appraisal politics in the workplace. Whereas prior studies have focused on turnover intention, employee performance, and deviant work behavior, this study proposes that importance should be given to CWB, because it has widespread and destructive consequences (Cohen and Diamant, 2019; Cohen and Özsoy, 2021; Searle, 2022) and can be a long-standing problem in the workplace. Although previous studies have considered the influence of organizational politics and performance justice on the CWB, to the best of our knowledge there has not been similar research on the relationship between performance appraisal politics and CWB. The present research magnifies the antecedent of CWB, and our study was also designed from the appraisee perspective, while prior studies on performance appraisal politics have focused on the appraiser perspective and overlooked the political behavior of coworkers and the organization’s political considerations (e.g., promotion, pay). As Tziner and Rabenu (2018) have argued, the appraisee’s reactions to the performance appraisal process are one of the indicators for effective performance formats. As the recipients of the rater’s decision, appraisees are more sensitive to the performance appraisal politics around them, and they are concerned with how the political behaviors of all performance appraisal participants (e.g., appraiser, coworker, and organization may affect their ratings. This study, therefore, clarifies that it is necessary to study performance appraisal politics from the appraisee’s perspective.

Second, we have verified the mediating role of appraisee POJ in the association of PAPP and CWB based on SCT. Performance appraisal politics essentially view the appraisal process as a political game (Kwon, 2020). Tziner and Rabenu (2018) has argued that rating accuracy is only important insofar as it affects employee motivation, mainly through employees’ perceptions of the fairness of the process. Ambiguity, uncertainty, and doubt infuse the appraisee’s experience of performance appraisal, and they have to ensure their own status and abilities by comparing their own ratings with those of others. Appraisees are thus extremely concerned about the ratings of fellow appraisees with similar abilities. This comparison process may invoke their POJ and resulting behavioral responses. Previous studies have generally relied on social exchange theory to explore how the appraiser’s political behavior in performance appraisal affects the appraisee’s attitudes and behaviors, and it has been reported that the appraisee tends to reciprocate the positive or negative behavior of the appraiser according to the ratings received (Rosen et al., 2017; Naseeb et al., 2019). However, we have found that negative organizational behaviors such as CWB are still prevalent among appraisees who benefit from performance appraisal politics, so social exchange theory only partially explains this phenomenon. As proposed in the present study, SCT broadens the theoretical explanation for the mechanism through which performance appraisal politics affects employees’ attitudes and behaviors.

Third, culture has been shown to affect how workers view performance appraisal (Tziner and Rabenu, 2018), and the existing literature also advocates considering PAPP as culture-driven (Dhiman and Maheshwari, 2013; Hofman and Newman, 2014), but little research has verified the mechanism through which performance appraisal politics affects employees’ attitudes and behaviors in a Chinese culture setting. In this study, we believe the influence of PAPP and CWB may be explained by the values of the Chinese collectivism culture. On the one hand, performance appraisals have lower relevance in collectivist national cultures (e.g., China, Japan, and Korea) than in individualist cultures, and thus often become conformance appraisals (Gerhart and Fang, 2005). In a high collectivist cultural setting, individuals who sincerely follow the orders and rules given above tend to receive high ratings, while those who prioritize individual performance and merit are socially seen as individuals who disrupt harmony from selfish motives (Kwon, 2020). In this regard, people immersed in a collectivist culture tend to highlight maintaining harmony with others (Lin and Sun, 2012; Fang et al., 2015; Wu et al., 2020), because they prefer a tightly knit framework in a society wherein individuals expect their relatives or in-group members to look after them in exchange for loyalty (Hofstede, 1989, 2001). Culture concomitants have also been shown to impinge on the way raters treat employee performance. Raters in a high-collectivist culture give more importance to team performance than to individual performance (Tziner and Rabenu, 2018). Thus, such culture contexts would be fertile ground for manipulating appraisal politics practices. On the other hand, prior research has concentrated primarily on the negative outcomes of performance appraisal politics and less attention has been directed to how to relieve this. Culture influences how people deal with conflicts (Wu et al., 2020). Appraisees with high PS may have political motives for conveying a socially desired self-image of harmony and integration to their appraisers to gain acknowledgment rather than to work solely on individual tasks and merits (Huo and Von Glinow, 1995). They thus indirectly influence the appraiser’s decisions in appraisal processes. Appraisees’ higher PS may present more salient strategic advantages in the appraisal game for a collectivist culture (Kwon, 2020). In addition, this study has indicated that face-saving for others is a special characteristic of PS among Chinese people, which is consistent with Confucius’s view of humaneness. As such, we think that individual PS has a particular role in dealing with performance appraisal politics in collectivist cultures. Overall, this study not only adds more culture evidence to the existing literature but also allows us to realize the importance of examining appraisal politics in different cultural settings.

### Practical implications

Our findings have several implications for managers and HR practitioners. First, the findings have confirmed that appraisee PAPP leads to lower POJ, which is accompanied by increasing CWB. When the appraisee thinks that performance appraisal is a tool to achieve the appraiser’s self-interest rather than truly assessing the appraisee’s performance, the healthy functioning of the organization may be damaged. Thus, the organization should pay significant attention to the negative outcome of performance appraisal politics and endeavor to reduce the incidence of CWB through a variety of strategies, such as providing an objective reflection of employee performance in the tasks of the specific job or position in the organization (Tziner and Rabenu, 2018); supporting a fair process that is the result of decision-making; applying performance supervision and feedback mechanisms to control the appraiser’s accountability and voice (Dhiman and Maheshwari, 2013); and hiring third-party performance appraisal services. Second, if performance appraisal politics cannot be effectively avoided, the organization can counteract its negative outcomes by strengthening or restoring employees’ POJ by, for example, inviting employees to participate in the design of the performance system, encouraging the employees to speak up for performance appraisal reform, keeping sustainable communication and feedback with the appraisee, and strengthening communication and feedback of performance results. Third, the finding that PS buffers the negative indirect impact of appraisee PAPP on CWB suggests that PS can at least offset the destructive consequences of performance appraisal politics. This highlights that enhancing PS is one way to diminish CWB. The organization may consider opting for process-focused training methods or programs to develop PS among their employees (Kaur and Kang, 2022). Organizations should also pay more attention to recruiting and promoting talents with high PS.

### Limitations and future research

There are several limitations in the current study that need to be acknowledged. First, social desirability might have affected the responses to self-reported CWB, although the single-level method was used to investigate employee emotion and behavior is more predictive than the cross-level method (Geertshuis et al., 2015). Additionally, due to the concealment and subjectivity characteristic of performance appraisal political behavior, there may be differences in cognition between the appraisee and the appraiser, so a future matched pair study on performance appraisal politics should be designed.

Second, the foregoing studies based on the appraiser motivation perspective have shown that performance appraisal politics exhibits both positive and negative effects on employee organizational behavior (Javed et al., 2013). We likewise think there could be a possible positive influence of performance appraisal politics on employee non-performance behavior because performance appraisal politics devotes increasing attention to non-performance considerations instead of giving sole attention to individual performance. This may be an interesting topic for future research. The vast majority of research has focused on the outcomes of performance appraisal politics, while little research has examined its antecedents. Future studies need to address this. A theoretical, conceptual model of behavior rating, including proximal contextual factors (rater attitudes and beliefs to the immediate task of appraising subordinates’ performance) and distal contextual factors (rater attitudes and beliefs about their organizations), orientation toward the performance appraisal system, and the rater’s personality characteristics, has been presented by Tziner and Rabenu (2018). In this model, these influences are discussed in more detail, which provides a more comprehensive understanding of performance appraisal politics from the rater’s perspective.

Third, high individual PS effectively alleviates the negative consequences of PAPP, because employees with such skills have a highly developed self-regulatory capacity (García-Chas et al., 2015; Xue et al., 2020) and the ability to influence others to achieve goals (Kranefeld et al., 2020). Previous studies have substantiated that subordinates with higher PS (e.g., social astuteness, interpersonal influence, and networking abilities) are likely to achieve more positive supervisor ratings (Kwon, 2020). This also raises the concern that such elevated PS may further intensify performance appraisal politics and weaken the basic function of performance appraisal. This is worth further discussion. Although the current study investigated the moderating role of PS, there may be other moderators requiring further exploration. For example, most studies have concluded that culture affects how organizations operate in their approach to performance appraisal systems, as well as raters’ attitudes and behaviors in response to employee performance appraisal (Fang et al., 2015; Tziner and Rabenu, 2018; Kwon, 2020; Wu et al., 2020). Tziner and Rabenu (2018) have argued that the appraiser evaluation in an organization scoring high in power distance will probably be the main indicator by which the employee will be rewarded with bonuses or promotion; however, in a low power distance culture, the employee’s performance appraisal is likely to be examined using objective parameters not dependent on the supervisor’s whim. Further analysis could be conducted to explore these cultural differences in appraisal politics.

## Data availability statement

The raw data supporting the conclusions of this article will be made available by the authors, without undue reservation.

## Ethics statement

The studies involving human participants were reviewed and approved by the Hubei Polytechnic University’s Human Research Ethics Committee. The patients/participants provided their written informed consent to participate in this study.

## Author contributions

Both authors listed have made a substantial, direct, and intellectual contribution to the work, and approved it for publication.
